# Negative religious coping is associated with fear of childbirth and pregnancy distress: findings from correlational and regression analyses

**DOI:** 10.1590/1806-9282.20252166

**Published:** 2026-06-29

**Authors:** Simge Evrenol Öçal, Şahika Şimşek Çetinkaya

**Affiliations:** 1 Izmir Katip Celebi University, Faculty of Health Science, Department of Obstetrics and Gynecology Nursing – İzmir, Türkiye.; 2 Kastamonu University, Faculty of Health Science, Department of Midwifery – Kastamonu, Türkiye.

**Keywords:** Anxiety, Childbirth, Fear, Pregnancy, Religion

## Abstract

**OBJECTIVE:**

The aim of this study was to examine the associations between positive/negative religious coping, fear of childbirth, and pregnancy-related distress among pregnant women.

**METHODS:**

A descriptive correlational design was used at a training and research hospital (June 2023–March 2024). A total of 155 pregnant women completed a demographic form, the Religious Coping Scale, the Tilburg Pregnancy Distress Scale, and the Wijma Delivery Expectancy/Experience Questionnaire. Analyses included descriptive statistics, Pearson correlations, t-tests, analysis of variance, and multiple regression.

**RESULTS:**

Participants reported higher positive than negative religious coping. Fear of childbirth was moderate, and pregnancy-related distress was low. In adjusted regression analyses, negative religious coping was associated with higher fear of childbirth and pregnancy-related distress (p<0.05), while positive coping was not significantly associated.

**CONCLUSION:**

Assessing religious coping in prenatal care may help identify women reporting greater childbirth fear and pregnancy-related distress. Spiritually sensitive support may be considered within holistic antenatal care. Longitudinal studies are needed to clarify the direction of these associations.

## INTRODUCTION

Pregnancy is a major life event involving profound anatomical, physiological, and psychosocial changes that shape women's roles and daily functioning^1^. Although it is a natural process, it is experienced differently by each woman and is influenced by both social and personal factors^2^. Fear of childbirth is characterized by uncertainty, anxiety, and avoidance related to labor and has been reported to affect 6–48% of pregnant women^3,4^. This fear often intensifies in late pregnancy and has been associated with longer and more complicated labor^5^.

Pregnancy also entails substantial psychological demands, including changes in body image, interpersonal relationships, and social roles, which may heighten anxiety and stress^6^. Elevated stress levels during pregnancy are linked to adverse maternal and fetal outcomes, including physiological complications and increased risks of morbidity and mortality^7,8^. Therefore, identifying factors that influence psychological distress and fear during pregnancy is essential for promoting maternal well-being.

One factor associated with emotional adjustment is religious coping^9,10^. Individuals frequently draw on religious beliefs when confronting uncertainty, stress, or a perceived loss of control^11^. While positive religious coping may foster resilience and emotional comfort, negative religious coping has been associated with poorer psychological well-being^12^. Religious beliefs can provide emotional stability and a sense of security^13^, suggesting that religious coping may influence how pregnant women manage childbirth-related fears and pregnancy-related distress. Although religious coping has been widely studied in relation to mental health, existing research has generally addressed religiosity as a broad construct and has primarily focused on its associations with sociodemographic and obstetric characteristics, childbirth self-efficacy, anxiety, and overall psychological well-being^10,14-16^. To date, no study has been identified that specifically examines the relationship between distinct forms of religious coping and fear of childbirth or pregnancy-related distress. Given these considerations, this study aimed to examine the relationships between positive and negative religious coping, fear of childbirth, and pregnancy-related distress among pregnant women. Addressing this gap may contribute to a more nuanced understanding of psychosocial factors associated with maternal distress and support the development of culturally sensitive prenatal care practices.

## METHODS

This descriptive correlational study was conducted with pregnant women attending the maternity clinic of a training and research hospital between June 2023 and March 2024. The study sample was selected using a purposive sampling method. The sample size was estimated using G*Power (effect size=0.30, power=95%, α=0.05), indicating a minimum of 138 participants. Women who met the following inclusion criteria were eligible to participate in the study: aged between 18 and 42 years, had a gestational age of ≥28 weeks, had no pregnancy-related risks (bleeding, risk of preterm labor, gestational diabetes, etc.), were primiparous, were literate in Turkish, had no psychiatric diagnosis, and provided informed consent to participate. A total of 187 women were invited to participate in the study. Of these, 155 women completed the survey, resulting in a response rate of 82.8%. Of the remaining 32 women, 19 initiated but did not complete the questionnaire (due to time constraints, being called for clinical procedures, or undergoing medical tests), and 13 declined participation. Data analysis was conducted using the responses of the 155 pregnant women who completed the study. No demographic data were collected from non-participants. Post-hoc power analysis confirmed sufficient statistical power for both regression models (fear of childbirth: f^2^=0.145, power=0.90; pregnancy distress: f^2^=0.103, power=0.80). Data were obtained through individual face-to-face interviews conducted in a private room, each lasting approximately 15–20 min.

The Personal İnformation Form examined sociodemographic, pregnancy, and religious coping characteristics of the pregnant women and consisted of 10 questions.

The Religious Coping Scale, developed by Eksi and Sayin^17^, includes 10 items across two subscales: Positive (7 items) and Negative Religious Coping (3 items). The scale uses a 4-point Likert format (1="hardly ever" to 4="frequently"), and subscales are scored separately. Possible scores range from 7 to 28 for positive coping and 3 to 12 for negative coping. In the original study, Cronbach's alpha values were 0.91 and 0.86^17^; in the present study, they were 0.85 and 0.89.

The Tilburg Pregnancy Distress Scale (TPDS), developed by Pop et al.^18^, assesses pregnancy-related distress and consists of 16 items across two subscales: Negative Affect (12 items) and Partner Involvement (4 items). Items are rated on a four-point Likert scale (0=very often, 1=quite often, 2=sometimes, 3=rarely/never), with several items reverse-scored (3, 5, 6, 7, 9, 10, 11, 12, 13, 14, 16). Total scores range from 0 to 48, with higher scores indicating greater distress. The Turkish validity and reliability study was conducted by Capik and Pasinlioglu, reporting a Cronbach's alpha of 0.83^19^. In the present study, Cronbach's alpha was 0.76.

The Wijma Delivery Expectancy/Experience Questionnaire Version A (W-DEQ A), developed by Wijma et al.^20^ and adapted into Turkish by Korukcu^21^, assesses fear of childbirth. The scale includes 33 items scored on a six-point Likert scale (0=completely to 5=not at all). Negatively worded items (2, 3, 6, 7, 8, 11, 12, 15, 19, 20, 24, 25, 27, 31) are reverse-scored. Total scores range from 0 to 165, with higher scores indicating greater childbirth fear. Fear levels are categorized as mild (≤37), moderate (38–65), severe (66–84), and clinical (≥85). The Turkish adaptation reported a Cronbach's alpha of 0.89^21^. In the present study, Cronbach's alpha was 0.73.

Statistical analyses were performed using SPSS 26.0. Normality was assessed through skewness and kurtosis (±1.5). Descriptive statistics summarized participant characteristics and scale scores. Differences across demographic variables were examined using independent samples t-test and one-way ANOVA. Pearson correlation analysis assessed associations among religious coping, pregnancy distress, and childbirth fear. Multiple linear regression analyses were performed to identify predictors of fear of childbirth and pregnancy distress, with model assumptions tested and satisfied. A significance level of p<0.05 was adopted for all analyses.

The study received approval from the Non-Interventional Clinical Research Ethics Committee (03/23/2023, No: 0137). Written and verbal informed consent was obtained from all participants, and the study was conducted in accordance with the Declaration of Helsinki.

## RESULTS

The mean age of participants was 25.47±5.18 years, with most aged 25–29 years. Half were high school graduates, 78.7% were unemployed, 50.3% reported income equal to expenses, and 69.7% had social security coverage. Most women were in the 28–40-week gestational range (67.7%), and 65.8% had planned pregnancies. Additionally, 68.4% reported engaging in religious practices when experiencing fear or sadness (Table 1).

**Table 1:** Characteristics of participants.

Variables		Number (n)	Percentage (%)
Age [Mean±SD (min–max)=25.47±5.18 (18–40)]			
	18–24	70	45.2
	25–29	53	34.2
	30–34	21	13.5
	35–40	11	7.1
Education level			
	Primary school or below	20	13.0
	Middle school	48	31.0
	High school	50	32.3
	Bachelor's degree or higher	37	23.7
Employment status			
	Employed	33	21.3
	Unemployed	122	78.7
Income level			
	Income less than expenses	47	30.3
	Income equal to expenses	78	50.3
	Income more than expenses	30	19.4
Social security status			
	Yes	108	69.7
	No	47	30.3
Planned pregnancy			
	Yes	102	65.8
	No	53	34.2
Engagement in religious behaviors during fear/sadness			
	Yes	106	68.4
	No	49	31.6

SD: standard deviation.

Regarding religious coping, the mean positive coping score (M=24.18, SD=4.18) was higher than the negative coping score (M=7.07, SD=3.28). The TPDS total score was 17.50±7.67, indicating low pregnancy-related distress, with subscale scores of 12.94±6.64 for Negative Affect and 4.55±3.71 for Partner Involvement. The mean W-DEQ score was 54.98±24.59, reflecting a moderate level of childbirth fear (Table 2). Correlation analyses showed a significant negative association between positive religious coping and fear of childbirth (r=-0.170, p=0.034), but no association with TPDS scores (p>0.05). Negative religious coping was significantly correlated with TPDS total scores (r=0.182, p=0.023) and the Partner Involvement subscale (r=0.206, p=0.010), but not with W-DEQ scores (p>0.05). Strong positive correlations were observed between TPDS and W-DEQ scores (r=0.586, p<0.001), and between both TPDS subscales and W-DEQ (p<0.001; Table 2).

**Table 2:** Comparison of mean scores of the Religious Coping Scale, Tilburg Pregnancy Distress Scale, and Wijma Delivery Expectancy/Experience Questionnaire.

Scales	Mean±SD			Min–max		
PRC	24.18±4.18			7–28		
NRC	7.07±3.28			3–12		
TPDS-T	17.50±7.67			0–41		
TPDS-NA	12.94±6.64			0–32		
TPDS-PI	4.55±3.71			0–15		
W-DEQ-T	54.98±24.59			6–111		
	**PRC**	**NRC**	**TPDS-T**	**TPDS-NA**	**TPDS-PI**	**W-DEQ-T**
PRC						
NRC	0.270**	-				
TPDS-T	-0.101	0.182*	-			
TPDS-NA	-0.044	0.095	0.875**	-		
TPDS-PI	-0.131	0.206*	0.500**	0.018	-	
W-DEQ-T	-0.170*	0.141	0.586**	0.448**	0.410**	

* p<0.05;

** p<0.01.

Note: PRC: positive religious coping; NRC: negative religious coping; TPDS-T: Tilburg Pregnancy Distress Scale total; TPDS-NA: Tilburg Pregnancy Distress Scale negative affect; TPDS-PI: Tilburg Pregnancy Distress Scale partner ınvolvement; W-DEQ-T: Total Wijma Delivery Expectancy/Experience Questionnaire Score; SD: standard deviation.

Multiple regression analyses were performed, controlling for sociodemographic and obstetric variables. For childbirth fear (W-DEQ), the model was significant, F(6, 167)=4.04, p<0.001, explaining 12.7% of the variance (adjusted R^2^=0.091). Negative religious coping (β=0.205, p=0.012) and engaging in religious behaviors during fear/sadness (β=0.208, p=0.012) were significantly associated with greater fear. For pregnancy distress (TPDS), the model was also significant, F(7, 166)=2.13, p=0.046, explaining 9.3% of the variance (adjusted R^2^=0.050). Only negative religious coping was associated with higher distress (β=0.234, p=0.005). Positive religious coping and other covariates were not significant in either model (Table 3). Overall, negative religious coping showed consistent associations with higher childbirth fear and pregnancy-related distress, while positive religious coping was not significantly associated with these outcomes.

**Table 3:** Multiple regression analysis considering the variables with acceptable significance predicting some characteristics, Wijma Delivery Expectancy/Experience Questionnaire, and Tilburg Pregnancy Distress Scale.

Dependent variable	Predictor	B	Coefficient standard error	β	t	p	Tolerance	VIF
Constant^a^		52.436	22.259		2.356	**0.020** *		
Wijma Delivery Expectancy/Experience Questionnaire	Positive religious coping	-0.864	0.500	-0.147	-1.726	0.086	0.813	1.230
	Negative religious coping	1.536	0.607	0.205	2.529	**0.012** *	0.899	1.112
	Age	-0.099	0.396	-0.021	-0.250	0.803	0.848	1.179
	Social security	-6.832	4.404	-0.128	-1.551	0.123	0.845	1.156
	Planned pregnancy	7.118	4.133	0.138	1.722	0.087	0.922	1.084
	Religious behavior (fear/sadness)	10.961	4.307	0.208	2.545	**0.012** *	0.884	1.131
Constant^b^		19.307	7.298		2.646	**0.009** *		
Tilburg Pregnancy Distress Scale	Positive religious coping	-0.268	0.160	-0.146	-1.674	0.096	0.807	1.240
	Negative religious coping	0.547	0.194	0.234	2.821	**0.005** *	0.898	1.114
	Age	-0.067	0.128	-0.045	-0.521	0.603	0.822	1.217
	Social security	-1.485	1.407	-0.089	-1.055	0.293	0.862	1.160
	Planned pregnancy	2.462	1.319	0.153	1.867	0.064	0.922	1.085
	Religious behavior (fear/sadness)	-0.267	1.375	-0.016	-0.194	0.847	0.883	1.133

a F=4.043, R^2^=0.127, p<0.05, Adjusted R^2^=0.091, Durbin-Watson=2.011.

b F=2.132, R^2^=0.093, p<0.05, Adjusted R^2^=0.050, Durbin-Watson=2.129.

* p<0.05.

Bold values indicate statistical significance at p<0.05. VIF: variance inflation factor.

## DISCUSSION

Individuals employ various strategies to cope with adverse or stressful life events, one of which is spiritual or religious orientation^16^. Religious coping involves interpreting difficulties from a religious perspective and assigning positive or negative meaning to these experiences. It is typically categorized into positive and negative dimensions: positive coping reflects a sense of spiritual connectedness through practices such as praying and seeking divine support, whereas negative coping includes feelings of abandonment, punishment, or viewing events as manifestations of evil^22^. Religious coping has been recognized as an influential strategy for managing stressful life events^11,16^.

Pregnancy is a critical period marked by extensive physical, emotional, and social changes, and women often adopt a range of coping strategies to manage associated stressors. Seeking spiritual or religious support is one of these strategies^9,10^. This study examined the relationship between religious coping, fear of childbirth, and pregnancy distress, contributing to a deeper understanding of how religious orientations shape psychological adjustment during pregnancy.

Findings indicated that participants used positive religious coping more frequently than negative coping. The mean childbirth fear score (M=54.98, SD=24.59) indicated a moderate level of fear, while pregnancy distress scores were relatively low. Similar studies in Türkiye have reported high levels of positive religious coping among pregnant women and have shown that negative coping correlates with greater stress, anxiety, and depressive symptoms^16,23,24^. Although international studies often use different instruments, evidence consistently suggests that the orientation of religious coping is linked to stress and anxiety levels during pregnancy^10,15^. Research also indicates that religiosity and spirituality are associated with improved psychological well-being, and that pregnant women who engage in religious coping tend to experience lower anxiety^16^. These findings support the notion that spiritual coping may serve as a protective factor during pregnancy.

Correlation analyses in this study showed that positive religious coping was associated with lower childbirth fear, whereas negative religious coping was related to higher pregnancy distress. Positive coping appears to strengthen emotional control and reduce fear, while negative coping may intensify guilt, helplessness, or perceptions of punishment, thereby increasing distress. A strong positive correlation between pregnancy distress and childbirth fear further suggests that general anxiety during pregnancy heightens childbirth-related concerns. Previous studies similarly indicate that pregnancy distress is a significant predictor of childbirth fear^25,26^. Given that pregnancy distress encompasses uncertainty about labor, concerns about fetal well-being, and perceived partner support, higher distress understandably amplifies childbirth-related anxiety. These results underscore the need for psychosocial support programs that address both childbirth fear and general stress management.

Regression analyses showed that negative religious coping and engaging in religious behaviors during fear or sadness were significantly associated with higher childbirth fear, while only negative religious coping was associated with pregnancy distress. Although negative religious coping was not significantly correlated with childbirth fear in the bivariate analysis, its association became evident in the multivariate model. This pattern may indicate a possible suppression effect, whereby controlling for related variables reveals the unique contribution of a predictor. In this study, age, social security status, pregnancy planning, and religious behaviors were considered simultaneously, and accounting for these overlapping influences clarified the independent association between negative religious coping and childbirth fear. These findings emphasize that the quality of religious coping—rather than its mere presence—shapes psychological outcomes. Consistent with previous research, negative religious coping may increase stress by reinforcing feelings of guilt, punishment, or spiritual struggle^22,23^. During the emotionally sensitive period of pregnancy, interpreting distress through punitive religious beliefs may be associated with heightened fear and anxiety. Moreover, religious behaviors do not always function as protective mechanisms; in certain contexts, they may inadvertently reinforce existing fears. At the same time, because of the cross-sectional design, it is not possible to determine the direction of this association. It is equally plausible that women experiencing greater fear are more likely to turn to religious behaviors as a coping response, rather than these behaviors leading to increased fear. Therefore, this finding should be interpreted as reflecting an association rather than a directional or causal relationship. In addition, religious behavior during fear or sadness was assessed with a single item, which may reflect a general tendency rather than a comprehensive coping construct; therefore, this finding should be interpreted with caution. The relatively low educational level and socioeconomic status of the sample may also have influenced how participants interpreted stressful experiences during pregnancy, possibly encouraging religious attributions. This sociocultural context may help explain why negative religious interpretations, such as perceiving distress as punishment or spiritual struggle, were linked to higher levels of psychological distress. Therefore, the relationship between negative religious coping and psychological distress should be understood within a broader sociocultural and economic framework. Although the regression models were statistically significant, the proportion of explained variance was modest. This suggests that religious coping represents only one of multiple factors associated with childbirth fear and pregnancy-related distress. Other relevant clinical and psychosocial variables—such as perceived social support, previous traumatic experiences, mental health history, or obstetric complications—may also contribute to these outcomes but were not included in the present models. Accordingly, religious coping should be considered as part of a broader psychosocial assessment that also addresses emotional, relational, and contextual influences.

These results highlight the importance of evaluating religious coping styles in prenatal care. Pregnant women's religious beliefs may influence their emotional responses, and counseling approaches that address maladaptive religious interpretations—such as guilt or perceived punishment—could be beneficial. Prenatal education and psychosocial programs should acknowledge women's religious and cultural values while addressing their psychological implications with sensitivity. Incorporating an assessment of religious coping into prenatal counseling may support personalized interventions. Increasing awareness of how religious beliefs influence coping may also help nurses and midwives provide culturally sensitive care. Identifying tendencies toward negative coping and promoting more supportive spiritual perspectives may reduce childbirth fear and enhance psychological well-being. In conclusion, this study demonstrates that religious coping significantly influences psychological adjustment during pregnancy. Recognizing the multidimensional nature of religious coping and incorporating it into prenatal counseling may strengthen efforts to reduce childbirth fear and promote maternal emotional well-being.

### Limitation

This study has several limitations. First, its cross-sectional design allows the examination of associations between religious coping, fear of childbirth, and pregnancy distress, but does not permit causal inferences. Longitudinal research is needed to clarify how these variables relate to one another over time. Second, the use of purposive sampling and the recruitment of participants from a single tertiary hospital in Western Türkiye limits the generalizability of the findings. The sample—composed largely of high school graduates and unemployed women—may not reflect the broader socioeconomic and cultural diversity of the pregnant population. In addition, the inclusion criteria may have resulted in a relatively psychologically healthier sample, potentially leading to an underestimation of distress and fear levels. Future studies employing random or stratified sampling across multiple regions would help strengthen external validity. Third, although the regression models were statistically significant, the explained variance was modest, indicating that many other psychological, social, and clinical factors not included in this study may influence fear of childbirth and pregnancy-related distress. Fourth, data were collected through face-to-face interviews in a clinical setting, which may have increased the risk of social desirability bias, particularly regarding religious behaviors and emotional experiences. Finally, although validated instruments were used, some internal consistency values were slightly lower than those reported in previous studies, which may have affected measurement precision. Additionally, the hospital's relocation during data collection hindered recruitment, reduced the anticipated sample size, and may have introduced selection bias. Nevertheless, the study achieved adequate statistical power for its primary analyses. Larger, prospectively recruited samples are recommended to verify and extend these findings.

## CONCLUSION

This study identified significant associations between pregnant women's religious coping styles, pregnancy-related distress, and fear of childbirth. Negative religious coping was associated with higher levels of both pregnancy distress and fear of childbirth, whereas positive religious coping showed no significant associations with these outcomes. These findings suggest that maladaptive or negatively interpreted religious cognitions may be linked to less favorable emotional experiences during pregnancy. However, the regression models explained a relatively limited proportion of the variance in psychological outcomes, indicating that religious coping represents only one aspect of a broader set of emotional, relational, and contextual factors influencing maternal distress. Therefore, the findings should be interpreted with caution and should not be taken to imply that religious coping alone determines psychological well-being during pregnancy.

From a clinical perspective, exploring how pregnant women interpret stressful experiences within a religious framework may contribute to more comprehensive psychosocial assessments in prenatal care. Antenatal education and psychosocial support programs may benefit from culturally and spiritually sensitive approaches that consider both supportive and potentially distress-related aspects of religious coping. Nevertheless, longitudinal and multi-center studies with more diverse samples are needed to better understand the direction and strength of these relationships.

## Data Availability

The datasets generated and/or analyzed during the current study are available from the corresponding author upon reasonable request.
